# [Retracted] miR-381 functions as a tumor suppressor by targeting ETS1 in pancreatic cancer

**DOI:** 10.3892/ijmm.2025.5702

**Published:** 2025-12-01

**Authors:** Guanen Qiao, Jing Li, Jun Wang, Zhaoyang Wang, Wei Bian

Int J Mol Med 44: 593-607, 2019; DOI: 10.3892/ijmm.2019.4206

Following the publication of the above article, a concerned reader drew to the Editor's attention that, in Fig. 1D, the 'SW1990' and 'Bxpc-3' data panels were overlapping, suggesting that these data were derived from the same original source where experiments showing different experimental conditions were intended to have been portrayed. In addition, further pairings of overlapping data panels were identified with the Ki67 assay data shown in Figs. 7E and the immunohistochemical data shown in Fig. 10C, suggesting that these figures had similarly been assembled incorrectly. Furthermore, four of the centrally placed flow cytometric plots featured in Fig. 5A appeared to be too similar in terms of the distribution of the data to be confident that these were all derived from independently performed experiments, and finally, some of the western blot data shown in Fig. 4B were strikingly similar to data which had already appeared in another paper, also published in *International Journal of Molecular Medicine*, that featured the same first author (Guanen Qiao).

In view of the number of different problems and potential anomalies identified with various of the figures in this paper, the Editor of *International Journal of Molecular Medicine* has decided that this paper should be retracted from the journal on account of an overall lack of confidence in the presented data. The authors were asked for an explanation to account for these concerns, but the Editorial Office did not receive a satisfactory reply. The Editor apologizes to the readership of the Journal for any inconvenience caused.

